# The Homeobox Transcription Factor NKX3.1 Displays an Oncogenic Role in Castration-Resistant Prostate Cancer Cells

**DOI:** 10.3390/cancers17020306

**Published:** 2025-01-18

**Authors:** Audris Budreika, John T. Phoenix, Raymond J. Kostlan, Carleen D. Deegan, Marina G. Ferrari, Kristen S. Young, Sean W. Fanning, Steven Kregel

**Affiliations:** 1 Department of Cancer Biology, Cardinal Bernardin Cancer Center, Stritch School of Medicine Health Sciences Division, Loyola University Chicago, 2160 South First Avenue Building 112, Room 205, Maywood, IL 60153, USA; abudreika@luc.edu (A.B.); jphoenix@luc.edu (J.T.P.); rkostlan@luc.edu (R.J.K.); cdeegan@luc.edu (C.D.D.); mferrari2@luc.edu (M.G.F.); kyoung17@luc.edu (K.S.Y.); sfanning@luc.edu (S.W.F.); 2 Integrated Program in Biomedical Science, Biochemistry, Molecular and Cancer Biology, Loyola University Chicago, Maywood, IL 60153, USA

**Keywords:** prostate cancer, androgen receptor, NKX3.1, castration resistance, enzalutamide, therapy resistance

## Abstract

Background/Objectives: Prostate cancer (PCa) is the second leading cause of cancer-related death in men. The increase in incidence rates of more advanced and aggressive forms of the disease year-to-year fuels urgency to find new therapeutic interventions and bolster already established ones. PCa is a uniquely targetable disease in that it is fueled by male hormones (androgens) that drive tumorigenesis via the androgen receptor or AR. Current standard-of-care therapies directly target AR and its aberrant signaling axis but resistance to these therapies commonly arises, and the mechanisms behind the onset of therapy-resistance are still elusive. Research has shown that even with resistant disease, AR remains the main driver of growth and survival of PCa, and AR target genes and cofactors may help mediate resistance to therapy. Here, we focused on a homeobox transcription factor that exhibits a close relationship with AR—NKX3.1. Though NKX3.1 is traditionally thought of as a tumor suppressor, it has been previously reported to promote cancer cell survival by cooperating with AR. The role of NKX3.1 as a tumor suppressor perhaps in early-stage disease also contradicts its profile as a diagnostic biomarker for advanced prostate cancer. Methods: We investigated the physical interaction between NKX3.1 and AR, a modulated NKX3.1 expression in prostate cancer cells via overexpression and knockdown and assayed subsequent viability and downstream target gene expression. Results: We find that the expression of NKX3.1 is maintained in advanced PCa, and it is often elevated because of aberrant AR activity. Transient knockdown experiments across various PCa cell line models reveal NKX3.1 expression is necessary for survival. Similarly, stable overexpression of NKX3.1 in PCa cell lines reveals an androgen insensitive phenotype, suggesting NKX3.1 is sufficient to promote growth in the absence of an AR ligand. Conclusions: Our work provides new insight into NKX3.1’s oncogenic influence on PCa and the molecular interplay of these transcription factors in models of late-stage prostate cancer.

## Simple Summary

The homeobox transcription factor NKX3.1 is essential for the development of the prostate and has long been thought of as a tumor suppressor. We hypothesize that much like the androgen receptor (AR), NKX3.1 plays a pro-differentiative, tumor suppressive role in early-stage disease and potentially acts as an oncogene in late-stage, AR-driven prostate cancer. Our results indicate that NKX3.1 is a critical co-factor for AR in prostate cancer cells, and knockdown leads to decreased cell survival, while overexpression is enough to promote growth in androgen depleted and AR-inhibited conditions. Finally, we suggest that NKX3.1 may promote oncogenic activity in late-stage disease through upregulation of genes driven by AR at selective androgen response elements. Altogether, these data indicate that NKX3.1 is necessary and sufficient for prostate cancer growth, and like AR, functions as an oncogene in a context-dependent manner.

## 1. Introduction

Prostate cancer (PCa) is the most common non-cutaneous malignancy for males in the United States, and it is expected to take the lives of over 35,000 individuals in 2024 [1]. The androgen signaling axis plays a fundamental role in all aspects of prostate biology. A nuclear hormone-responsive transcription factor known as the androgen receptor (AR) is a master regulator of prostate epithelial cell function, survival, and differentiation [2]. During the development of PCa, AR undergoes a functional switch toward oncogenic activity; this can often be successfully antagonized with treatments such as systemic androgen deprivation, androgen synthesis inhibitors, or other direct AR-targeted therapies [3,4,5,6]. However, acquired resistance to these treatments is extremely common, which ultimately leads to progressive, metastatic disease [7,8].

AR interacts with many other transcription factors, including its pioneering cofactor Forkhead box A1 (FOXA1) and a specific NK class homeobox member, NKX3.1, to coordinate many aspects of prostate luminal cell biology [9,10]. NKX3.1 is necessary for the terminal differentiation of normal prostate epithelial cells [11], and its expression is largely confined to prostate tissue, concomitant with its regulation by androgens [12,13]. Although NKX3.1 is functionally well defined during prostate gland development, its contributions to PCa initiation and progression are debated; multiple lines of conflicting evidence exist as to whether NKX3.1 is permissive or suppressive to oncogenic activity.

Several studies have asserted that NKX3.1 is protective against oncogenesis. Genetically, the *NKX3-1* gene is located at chromosome 8p21, which undergoes frequent loss of heterozygosity (LoH) in PCa [14]. Functional studies revealed that whole-body mutant mice lacking one or both alleles (*NKX3-1*^+/−^, *NKX3-1*^−/−^) possess either atypical prostate morphology or epithelial dysplasia/hyperplasia [15]. Additional murine studies using prostate-specific conditional *NKX3-1* knockout systems (*NKX3-1*^flox/flox^) alone or with concurrent *PTEN* deletion resulted in a phenotype resembling prostatic intraepithelial neoplasia (PIN) [16,17]; precursor lesions that are seen in patients prior to onset of adenocarcinoma [18]. As such, this has prompted many suggestions that NKX3.1 functions as a tumor suppressor in prostate luminal cells.

However, many other factors suggest NKX3.1 might behave in an oncogenic manner. While *NKX3-1* ablation in the above mouse models resulted in abnormal morphology of the prostate and representative PCa precursor lesions, there was no overt progression to adenocarcinoma after prolonged monitoring [15,16,17]. Though there are inherent shortcomings of existing murine models of prostate cancer, it stands to reason that loss of *NKX3-1* alone is not sufficient to initiate PCa in mice [19].

Despite the commonly observed LoH in patients, wild-type NKX3.1 expression is almost always maintained in advanced disease, and, at times, even elevated directly due to AR activity [20]. The loss of one copy of NKX3.1 may be attributed to a whole chromosomal arm (8p) deletion that allows gain of the *MYC* oncogene frequently seen in prostate cancer at 8q [21]. This, together with evidence that gain of the *MYC* oncogene correlates with poor patient outcomes while NKX3.1 deletion does not [22], suggests that 8p21 deletion is truly the consequence of MYC amplification rather than a direct consequence of tumorigenesis. Despite assertions that it exerts a tumor-suppressive role in PCa, NKX3.1 has become an excellent and widely used IHC biomarker for metastasis of prostatic origin; it is even more reliable than traditional AR or prostate-specific antigen (PSA) staining [23]. Altogether, the role of NKX3.1 in PCa is widely contested; more research is needed to define its function as it relates to traditional AR activity and targeted-therapy resistance.

The aim of this study is to clarify the functional role of NKX3.1 in PCa cells under varying contexts of AR activity, including androgen deprivation, androgen stimulation, and active antagonization in the form of the commonly clinically-utilized AR-antagonist enzalutamide, in both therapy-naïve and therapy-resistant cells [8].

## 2. Materials and Methods

### 2.1. Cell Lines and Cell Viability

R1881 was sourced from Sigma-Aldrich (St. Louis, MO, USA), and enzalutamide (MDV3100) was sourced from Selleck Chemicals (Houston, TX, USA) and stored at −80 °C in DMSO. VCaP and CWR-22-Rv1 (22Rv1) cells were purchased from ATCC (Manassas, VA, USA). CWR-R1, VCaP, LNCaP, and enzalutamide-resistant counterparts in addition to BPH1, 957E/hTERT, NCI-H660 (H660), PC3, HEK293, DU145, PNT-2, and RWPE1 cells were generously provided by Dr. Donald J. Vander Griend at the University of Illinois in Chicago and have been previously characterized and cultured as described [8,24,25]. All cultures were routinely screened for the absence of mycoplasma contamination using an ATCC Universal Mycoplasma Detection Kit (Manassas, VA, USA). Lentiviral NKX3.1 overexpression constructs pLV[Exp]-Puro-CMV>3xFLAG/hNKX3-1[ORF009158] and pLV-GFP control were purchased from Vector Builder (Chicago, IL, USA). Cell viability was evaluated after seeding at 2500 cells per well in a 96-well plate with CellTiter-Glo^®^ Luminescent Cell Viability Assay (Promega, Madison, WI, USA) at 560 nM with the Synergy H1 Plate Reader (BioTek, Winooski, VT, USA).

### 2.2. Western Blots

Whole-cell lysates were collected from cells seeded at 1 × 10^6^ cells per well in a 6-well plate, lysed with RIPA-PIC buffer [150 mM sodium chloride, 1.0% Igepal CA-630 (Sigma-Aldrich, Burlington, MA, USA), 0.5% sodium deoxycholate, 0.1% SDS, 50 mM Tris, pH 8.0, 1x protease inhibitor cocktail (Roche Molecular Biochemicals; Penzberg, Germany)], scraped, and sonicated (Fisher Scientific, Hampton, NH; model FB-120 Sonic Dismembrator). Protein level was quantified by a BCA assay (Thermo-Fisher Scientific, Waltham, MA, USA), and 20–30 µg of protein was loaded per lane. Antibodies used: anti-AR (D6F11 XP^®^, Cell Signaling Technology, (Danvers, MA, USA)); anti-Beta Actin (AC-15, Sigma-Aldrich); anti-FOXA1 (3A8 PIMA1091, Thermo Fisher); anti-NKX3.1 [D2Y1A XP^®^, Cell Signaling Technology, (Danvers, MA, USA)]; anti-Glyceraldehyde-3-phosphate dehydrogenase (GAPDH) (Cell Signaling, Cat. #2118); and anti-cleaved PARP [Asp214 D64E10 XP^®^, Cell Signaling Technology, (Danvers, MA, USA)]. Secondary antibodies and Nitrocellulose membranes from Licor (Lincoln, NE, USA) were used and data were visualized using Licor Odyssey M system (Lincoln, NE, USA).

### 2.3. Knockdown with siRNA

Transient knockdown was performed using Silencer^®^ Select siRNAs (Invitrogen, Waltham, MA, USA) siNKX3.1 #1 (s9574, Sense Sequence 5′-3′: CAGCUAUCCUUACUACCCAtt) and siNKX3.1 #2 (s9575, Sense Sequence 5′-3′: GCUAUAAGACUAAGCGAAAtt) (see Supplementary Figure S1 for details), as well as Silencer^®^ Select Negative Control No. 1 siRNA (siNSC, 4390843) and Silencer^®^ Select Positive Control GAPDH (siGAPDH, 4390849). siRNAs were transfected into cells using Lipofectamine™ RNAiMAX (Invitrogen) according to manufacturer’s protocols. Cells were seeded at 2500 cells per well in a 96-well plate for cell viability analysis and 500,000 cells per well in a 6-well plate

### 2.4. Quantitative Reverse Transcription PCR (Q-RT-PCR)

RNA was purified from similar growth conditions described above using the Qiagen RNeasy Mini Kit (Qiagen, Valencia, CA, USA), and quality was tested using the NanoDrop 2000 Spectrophotometer (Thermo Fisher Scientific). Extracted RNA was converted to cDNA using an iScript cDNA Synthesis Kit (BioRad, Hercules, CA, USA). Levels of AR (Exons 1-2, Exon 4), AR-Variant-7 (AR-V7), *KLK3* [Prostate Specific Antigen (PSA)], *TMPRSS2* (Transmembrane protease serine-2), *NKX3-1* (NK3 homeobox 1, NKX3.1), *ERG* (ETS-related gene), Insulin-like growth factor binding protein-3 (*IGFBP3*), *C1orf116* [Specifically Androgen-Regulated Gene (SARG)], *NR3C1* [Glucocorticoid Receptor (GR)], and *FKBP5* (FK506 binding protein 5, FKBP prolyl isomerase 5) transcripts were quantified using Fast SYBR^®^ Green Master Mix (Invitrogen) using custom primers (see Table 1 for primer sequences). Standard curves were used to verify primer efficiency, and average change in threshold cycle (ΔCT) values were determined for each sample relative to endogenous β-actin (*ACTB*) and compared to siNSC control (ΔΔCT).

### 2.5. Co-Immunoprecipitation

Co-immunoprecipitation was performed on cells seeded as described above using a Dynabeads Protein G IP Kit (Invitrogen). Cells were lysed with CellLytic TM Lysis Reagent (Sigma Aldrich) and sonicated with the BioRupter^®^ Pico Sonicator (Diagenode, Denville, NJ, USA) for 10 cycles of 30 s on and 30 s off. Proteins were immunoprecipitated with Protein G dynabeads with anti-AR (PG-21 (Millipore, Burlington, MA, USA)), anti-FOXA1 (PA5-27157, Thermo Fisher), and anti-IgG (Normal Rabbit 12-370, Millipore Sigma). Proteins were eluted in 2.5x Laemmli Buffer and loaded onto 10% acrylamide gels and analyzed via Western blot.

### 2.6. NanoBiT Assay

The NanoBiT^®^ (NanoLuc^®^ Binary Technology, Naperville, IL, USA) PPI Starter Kit (Promega) was used for proximity assays, and pcDNA3.1 vectors were developed with BiT-tagged proteins of interest purchased from GeneScript Biotech (Piscataway, NJ, USA). Plasmids were isolated and purified. Transfection was performed with Lipofectamine ™ 3000 (Thermo Fisher).

HEK293 cells were plated at 9000 cells per well in a 96-well plate and transfected to a final plasmid concentration of 100 ng/well. Luminescence was measured on the BioTek BioSpa Live Cell Analysis System (Agilent, Santa Clara, CA, USA), and background signal was subtracted. Treatments were performed in triplicate with standard deviation of the mean for error.

### 2.7. Statistical Analysis

Data collected were analyzed using GraphPad Prism software version 9.0 f (GraphPad Software, La Jolla, CA, USA). Representative qPCR experiments were chosen for each gene of interest and performed in triplicate to determine mean standard error, and Student’s *t*-tests was used to ascertain statistical significance with ΔCt values compared to the control. Fold change (2^−ΔΔCt^) was normalized to siNSC, and cells were grown in normal growth conditions. A representative experiment was chosen for cell viability data with five technical replicates used to determine standard error. A Student’s *t*-test was performed to determine significance. A two-way ANOVA test was performed to obtain *p*-values for analysis across cell lines (siRNA knockdown) and treatment groups (vehicle DMSO and enzalutamide) at the day 5 timepoint.

## 3. Results

### 3.1. NKX3.1 Is Expressed in Prostate Adenocarcinoma and Forms a Complex with AR and FOXA1

**Figure 1:**
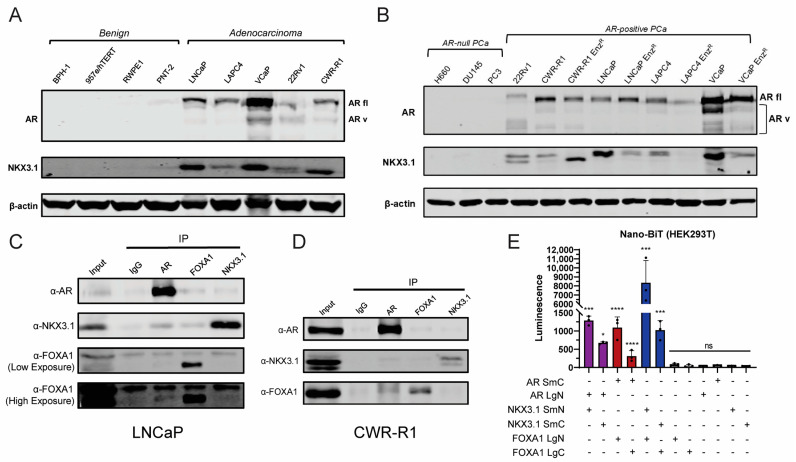
Expression levels of NKX3.1 vary across PCa cell lines and closely track with AR expression levels. (**A**) Western blot illustrating AR, NKX3.1, and B-actin protein levels in benign prostate cell lines and adenocarcinoma prostate cell lines. (**B**) Western blot illustrating protein levels of AR and NKX3.1 in AR-null cell lines and AR-positive cell lines. (**C**) Co-immunoprecipitation Western blot of LNCaP cells with IgG, AR, FOXA1, and NKX3.1 pulldown. (**D**) Co-immunoprecipitation Western blot of CWR-R1 cells with IgG, AR, FOXA1, and NKX3.1 pulldown. (**E**) Nano-BiT luminescence data depicting binding of large BiT (Lg) and small (Sm) BiT partners on constructs tagged on N- or C-terminally expressed AR, NKX3.1, and/or FOXA1. Significance was determined via one-way ANOVA comparing signal to each individual single BiT. The threshold for statistical significance set as follows: non-significant (ns) *p* > 0.05, * *p* < 0.05, *** *p* < 0.001, **** *p* < 0.0001.

We first assessed the expression of NKX3.1 and the AR protein in a panel of non-transformed (benign) and AR-positive adenocarcinoma cell lines (Figure 1A). NKX3.1 protein expression mirrors AR expression in these cells. The protein levels of AR and NKX3.1 exhibit a direct relationship and track with each other in a panel of AR-null and AR-positive PCa cell lines (Figure 1B). This is further bolstered by the fact that the AR-null cell lines have virtually no detectable expression of NKX3.1. Enzalutamide-resistant (Enz^R^) derivatives that show decreased AR expression, such as LAPC-Enz^R^, also show concurrent decrease in NKX3.1 levels (Figure 1B). We also see different sized bands of NKX3.1 across lines, suggesting expression of different isoforms (Figure 1B, see Supplemental Figure S1 for known isoforms). AR and its pioneering cofactor FOXA1 have been shown to interact with each other on the chromatin in prostate cancer cells [9], and we confirm a direct physical interaction of NKX3.1 with both AR and FOXA1 through co-immunoprecipitation in LNCaP (Figure 1C) and CWR-R1 (Figure 1D) cells, as well as through a Nano-BiT split luciferase (Promega) interaction system (Figure 1E). N-terminal tagged AR interacts strongly with both NKX3.1 and FOXA1, particularly in their N-termini, and the N-terminal-tagged NKX3.1 and FOXA1 interact with each other more strongly in this system, further confirming the interaction of all of these proteins at their N-termini (Figure 1E) [9].

### 3.2. NKX3.1 Knockdown Decreases Viability in All AR-Positive Cell Lines, Both Enzalutamide-Naïve and Enzalutamide-Resistant

**Figure 2:**
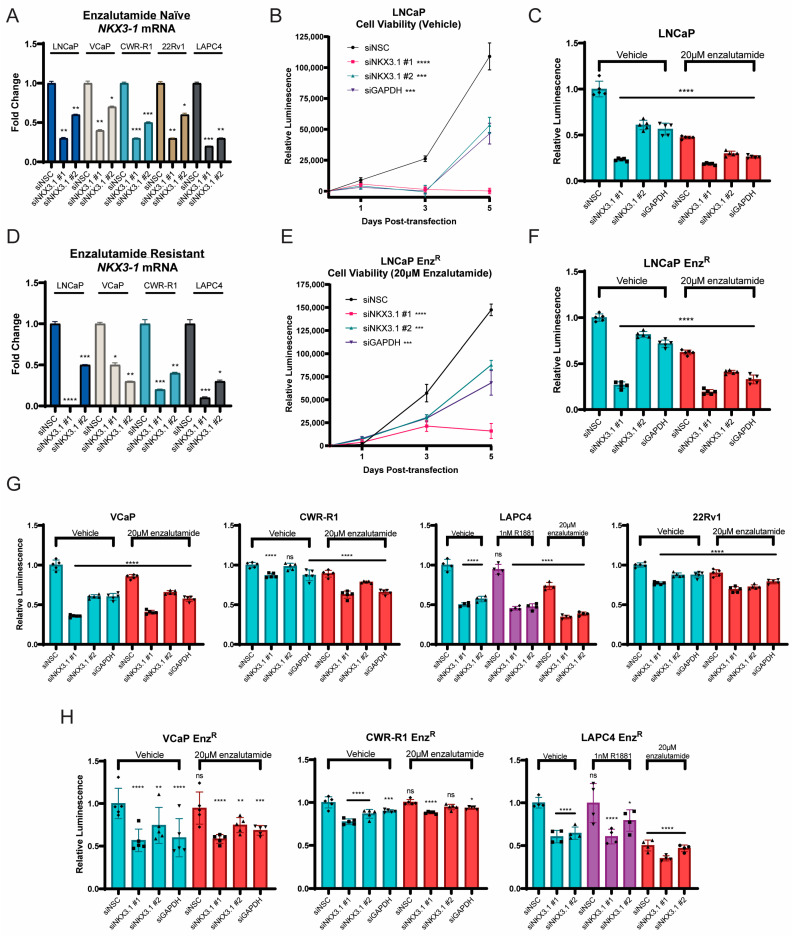
NKX3.1 is necessary for cell survival in enzalutamide-naïve and enzalutamide-resistant PCa cell lines. (**A**) NKX3.1 mRNA levels 72 h post-transfection in enzalutamide-naïve PCa cell lines. Statistical significance was determined with Student’s *t*-test relative to siNSC per cell line. (**B**) Cell viability of LNCaP cells treated with vehicle over a 5-day time course. Statistical significance was evaluated with one-way ANOVA relative to siNSC control at day 5. (**C**) Cell viability of LNCaP cells treated with vehicle or 20 µM enzalutamide at post-transfection day 5. Statistical significance was determined by two-way ANOVA and relative to siNSC vehicle control. (**D**) NKX3.1 mRNA levels 72 h post-transfection in enzalutamide-resistant PCa cell lines. Statistical significance determined with Student’s *t*-test relative to siNSC per cell line. (**E**) Cell viability of LNCaP Enz^R^ cells treated with 20 µM enzalutamide (normal growth conditions) over a 5-day time course. Statistical significance was evaluated with one-way ANOVA relative to siNSC control at day 5. (**F**) Cell viability of LNCaP Enz^R^ cells treated with vehicle or 20 µM enzalutamide at post-transfection day 5. Statistical significance was determined by two-way ANOVA and relative to siNSC vehicle control. (**G**) Cell viability of enzalutamide-naïve PCa cell lines treated with vehicle or 20 µM enzalutamide measured at post-transfection day 5. Statistical significance was determined by two-way ANOVA and relative to siNSC vehicle control for each cell line. (**H**) Cell viability of enzalutamide-resistant PCa cell lines treated with vehicle or 20 µM enzalutamide measured at post-transfection day 5. Statistical significance was determined by two-way ANOVA and relative to siNSC vehicle control for each cell line. The threshold for statistical significance set as follows: ns *p* > 0.05, * *p* < 0.05, ** *p* < 0.01, *** *p* < 0.001, **** *p* < 0.0001.

We next wanted to examine how depletion of NKX3.1 mRNA would affect the growth and viability of NKX3.1-positive cell lines. We obtained substantial knockdown of NKX3.1 by using two different siRNAs that target all known isoforms of NKX3.1 (Figure 2) [Silencer^®^ Select siRNA (Invitrogen) siNKX3.1 #1 (s9574), siNKX3.1 #2 (s9575), See Supplemental Figure S1 for target sites on the NKX3.1 transcript]. On the transcript level, siNKX3.1 #1 leads to a ~70–100% knockdown across cell lines, with siNKX3.1 #2 showing a ~40–80% knockdown efficiency, when compared to non-silencing control (siNSC) (Figure 2A,D). Knockdown efficiency was also similar in both enzalutamide-naïve (enzalutamide-sensitive LNCaP, VCaP, CWR-R1 and LAPC-4 and the de novo-resistant 22Rv1 [6]) (Figure 2A) and -resistant (LNCaP-Enz^R^, VCaP-Enz^R^, CWR-R1-Enz^R^, and LAPC-4 -Enz^R^ derived from the isogenic matched enzalutamide-sensitive lines, cultured long-term with enzalutamide [8]) (Figure 2D) cell lines. We then assessed viability in cells in our NKX3.1-positive cell lines. Both enzalutamide-naïve LNCaP (Figure 2B) and enzalutamide-resistant LNCaP-Enz^R^ (Figure 2E) cells displayed decreased viability and proliferation over the course of 5 days (viability at 5 days illustrated in Figure 2C–F), with the more efficient knockdown of siRNA #1 illustrating a greater decrease in viability, and siRNA #2 producing an effect like what is seen with positive control *GAPDH* siRNA knockdown (see Supplemental Figure S2 for confirmation of GAPDH knockdown in LNCaP and LNCaP-Enz^R^). These effects were reproduced across every AR-positive cell line we tested, both enzalutamide-naïve and -resistant, and an additive effect was seen with enzalutamide treatment (Figure 2G–H, Supplemental Figure S3 for proliferation over 5 days for 22Rv1, CWR-R1, CWR-R1-Enz^R^, VCaP, VCaP-Enz^R^, LAPC-4, and LAPC-4-Enz^R^). This growth inhibition coincided with increased PARP cleavage (cPARP), a readout of apoptosis [26] (See Supplemental Figure S4A).

### 3.3. NKX3.1 Knockdown Decreases AR Protein and AR Target Gene Expression in Enzalutamide-Naïve and Enzalutamide-Resistant Prostate Cancer Cells

**Figure 3:**
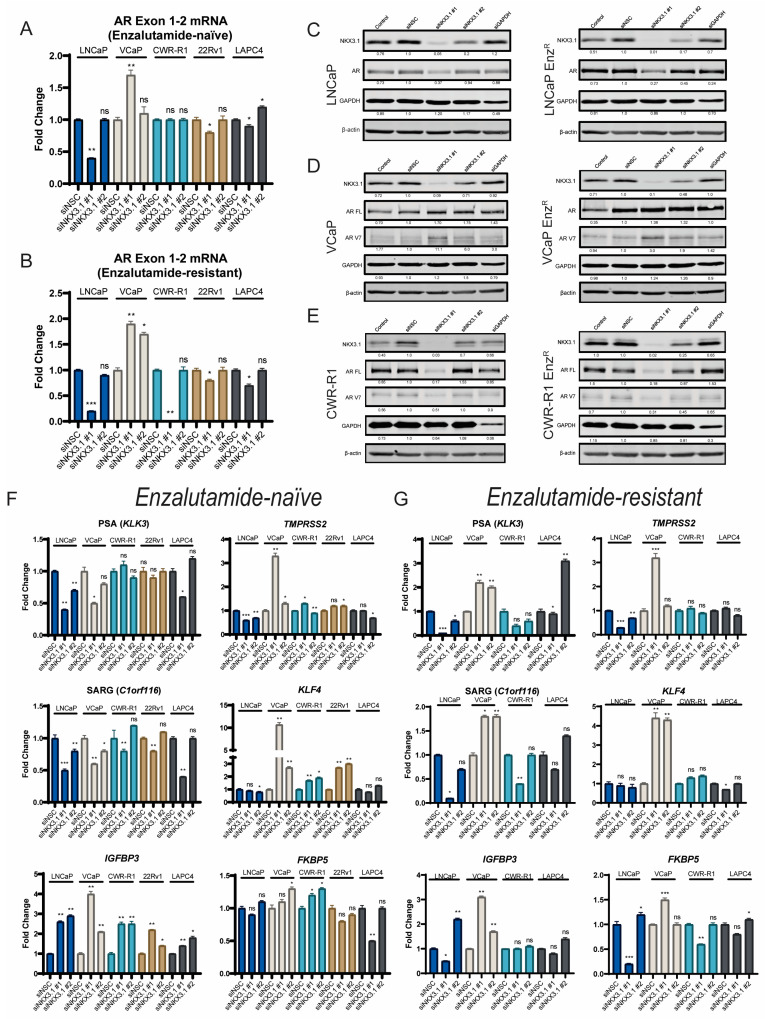
NKX3.1 knockdown reveals depletion of AR protein levels and decreases in AR target gene transcript levels. (**A**) Full-length AR mRNA levels (Exon 1-2) in enzalutamide-naïve cell lines. Statistical significance was determined with Student’s *t*-test and relative to siNSC control. (**B**) Total AR mRNA levels (Exon 1-2) in enzalutamide-resistant cell lines. Statistical significance was determined with Student’s *t*-test and relative to siNSC control. (**C**) Western blot of LNCaP and LNCaP Enz^R^ cells’ protein levels harvested 72 h post-transfection. Protein bands quantified relative to β-actin loading control. (**D**) Western blot of VCaP and VCaP Enz^R^ cells’ protein levels harvested 72 h post-transfection. Protein bands quantified relative to β-actin loading control. (**E**) Western blot of CWR-R1 and CWR-R1 Enz^R^ cells’ protein levels harvested 72 h post-transfection. Protein bands quantified relative to β-actin loading control. (**F**) Panel of AR target gene levels in enzalutamide-naïve cell lines. Statistical significance determined by Student’s *t*-test relative to siNSC within each cell line. (**G**) Panel of AR target gene levels in enzalutamide-resistant cell lines. Statistical significance determined by Student’s *t*-test relative to siNSC within each cell line. The threshold for statistical significance set as follows: ns *p* > 0.05, * *p* < 0.05, ** *p* < 0.01, *** *p* < 0.001.

Given NKX3.1’s physical interaction with AR and the role of AR as a driving oncogene both before and after enzalutamide resistance, we examined how NKX3.1 knockdown affects AR transcript, protein, and downstream target gene expression. NKX3.1 knockdown decreased AR transcript expression in most cell lines, both in enzalutamide-naïve (Figure 3A) and -resistant lines (Figure 3B) except for VCaP and VCaP-Enz^R^, which we and others have shown before responds to AR inhibition by upregulating the AR protein and transcript [6,8]. We investigated this on the protein level and find that NKX3.1 knockdown decreases NKX3.1 protein levels to a similar extent as what we see with the mRNA transcript, and the responses to what we see in AR mRNA transcript is recapitulated in protein via Western blot (Figure 3C–E). We also find that siRNA #1, which nearly abolishes NKX3.1 protein expression, leads to dramatic decreases in AR protein in LNCaP and LNCaP-Enz^R^ (Figure 3C), and lower NKX3.1 knockdown efficiency by siRNA #2 leads to smaller effects on the AR protein. The effect was similar in CWR-R1 and CWR-R1-Enz^R^, which also express the AR-variant AR-V7 (Figure 3E) implicated in enzalutamide resistance [27], and in isogenic 22Rv1, we see that full-length AR is decreased while AR-V7 expression is increased [Supplemental Figures S4B and S5 for AR-Exon 4 (full length) and AR-V7 mRNA levels]. We also observed similar effects in LAPC-4 and their matched enzalutamide-resistant counterpart, LAPC-4 Enz^R^ (Supplemental Figures S4C and S4D, respectively).

Furthermore, in the VCaP cell lines, we see a compensatory increase in AR expression and AR-V7, which occurs in VCaP when AR is inhibited [8]. To examine the downstream effects of the NKX3.1 and AR depletion, we examined known AR target genes in the enzalutamide-naïve (Figure 3F) and-resistant (Figure 3G) contexts and observe that broadly, when AR is downregulated, AR target genes are decreased across cell lines, except in VCaP where they are increased (see Supplemental Figure S2C,D for *ERG* expression in VCaP and VCaP-Enz^R^, which contain a TMPRSS2–ERG translocation [28]). Furthermore, we also identified GR upregulation in CWR-R1 cells with NKX3.1 knockdown (Supplemental Figure S2E), another hallmark of AR inhibition in these cells [29]. Taken together, these data illustrate how NKX3.1 supports AR expression and activity, and NKX3.1 knockdown mirrors the responses seen during AR inhibition [6,8] across different AR-positive cell lines.

### 3.4. NKX3.1 Overexpression Is Sufficient to Promote Growth During Androgen Depletion and AR-Antagonization in Enzalutamide-Naïve Prostate Cancer Cells and Upregulates Selective Response Element (sARE) Regulated AR Target Genes

**Figure 4:**
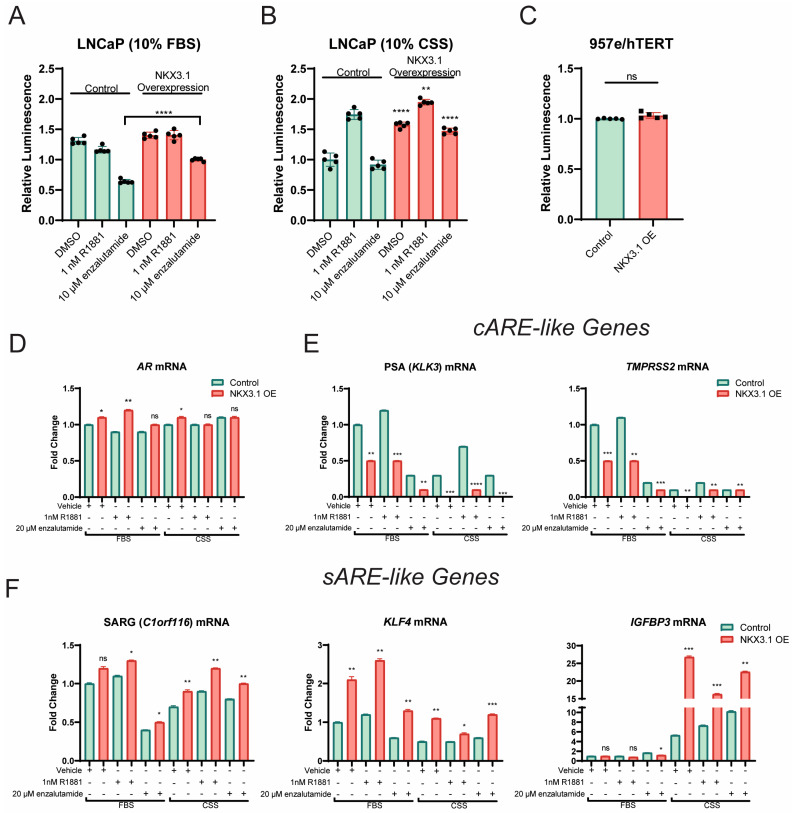
NKX3.1 overexpression blunts the effect of enzalutamide, promotes castration resistance, and upregulates sARE-like genes while downregulating cARE genes. (**A**) Cell viability at day 5 of LNCaP parental and 3x-Flag-tagged-NKX3.1-expressing LNCaP cells grown in 10% FBS and treated with vehicle DMSO, 1nM R1881, or 10 µM enzalutamide. Statistical significance determined by two-way ANOVA across treatment groups. (**B**) Cell viability at day 5 of LNCaP parental and 3x-Flag-tagged-NKX3.1-expressing LNCaP cells grown in 10% CSS and treated with vehicle DMSO, 1nM R1881, or 10 µM enzalutamide. Statistical significance determined by two-way ANOVA across treatment groups. (**C**) Cell viability at day 5 of 957e/hTERT cells expressing endogenous NKX3.1 and 3x-Flag-tagged NKX3.1. Statistical significance determined by two-way ANOVA. (**D**) AR mRNA levels (Exon 1-2) of LNCaP cells with endogenous NKX3.1 and LNCaP cells with 3x-Flag-tagged NKX3.1 at the day 3 timepoint grown in 10% FBS or 10% CSS and treated with vehicle DMSO, 1 nM R1881, or 10 µM enzalutamide. Statistical significance determined by two-way ANOVA across treatment groups. (**E**) mRNA levels of cARE-like genes at day 3 grown in 10% FBS or 10% CSS and treated with vehicle DMSO, 1 nM R1881, or 10 µM enzalutamide. Statistical significance determined with two-way ANOVA across each treatment group. (**F**) mRNA levels of sARE-like genes at day 3 grown in 10% FBS or 10% CSS and treated with vehicle DMSO, 1 nM R1881, or 10 µM enzalutamide. Statistical significance determined with two-way *ANOVA* across each treatment group. The threshold for statistical significance is set as follows: ns *p* > 0.05, * *p* < 0.05, ** *p* < 0.01, *** *p* < 0.001, **** *p* < 0.0001.

Given the necessity of NKX3.1 for cell viability, regardless of resistance to AR antagonists, in promoting AR expression and downstream AR target gene activity, we investigated how NKX3.1 overexpression affects proliferation and responses to AR inhibition. We overexpressed NKX3.1 in LNCaP cells and examined its effects compared to control cells under treatments to mimic common therapeutic strategies employed in the clinic: growth in phenol-red free media containing charcoal stripped serum (CSS), which is utilized to model the use of ADT therapy in treating PCa, and treatment with either physiological levels of androgens (1 nM R1881 [30]) or AR- ntagonization (20 μM enzalutamide [8]). We first examined growth in media containing 10% FBS with exogenous R1881 and enzalutamide over the course of 5 days (Supplemental Figure S6A,B). After 5 days of growth, there were modest differences between the control and overexpressed cell lines across all three treatments in 10% FBS media (Figure 4A), but most strikingly, overexpression of NKX3.1 blunted the growth inhibition seen with enzalutamide treatment in control cells. When cells were starved of basal androgens in CSS, baseline growth in the vehicle Dimethyl Sulfoxide (DMSO) treatment group was significantly elevated in NKX3.1-overexpressed LNCaP compared to control, suggesting a growth advantage in the absence of androgens (Figure 4B). Treatment with additional androgens with R1881 revealed larger increase in cell viability with overexpression compared to parental, and AR antagonization with enzalutamide revealed a decline in efficacy to reduce viability with the overexpression compared to control (Figure 4B). When NKX3.1 was overexpressed in AR-negative, immortalized prostate epithelial cells, 957E/hTERT, no growth advantage was seen (Figure 4C). Taken together, these data suggest that NKX3.1 overexpression in AR-positive cells is sufficient to promote survival in the absence of androgens and to promote resistance to AR antagonism by enzalutamide.

To investigate how NKX3.1 may be affecting the AR signaling axis, we first examined how NKX3.1 overexpression affected AR transcript (Figure 4D) and protein levels (Supplemental Figure S6C) and found very modest increases with NKX3.1 overexpression. We then wanted to investigate some of the functional consequences of NKX3.1 overexpression and AR upregulation by interrogating several commonly investigated AR target genes. Previously, we identified that some AR target genes are preferentially driven from consensus androgen response elements (cAREs), which are 6 base pair (bps) inverted repeats of 5′-AGAACA-3′, with 3 bps intervening [31], versus selective androgen response elements (sAREs), which are half-sites of the cAREs [32], that we identified as enriched by flanking FOXA1 and NKX3.1 motifs [33]. Canonical cARE-driven genes like PSA (*KLK3*) and *TMPRSS2* actually show downregulation with NKX3.1 overexpression, while still being driven by AR activation through R1881 (Figure 4E), while expression of sARE-driven genes, such as *IGFBP3*, SARG (*C1orf116*), and *KLF4* are increased with NKX3.1 overexpression (Figure 4F). This is concordant with our previous identification of NKX3.1 motifs enriched near the AR binding sites at these genes [33]. These data support the role of NKX3.1 in mediating AR-driven gene expression when bound to AR and co-localized on the chromatin.

## 4. Discussion

Taken together, our work presented here suggests the necessity of NKX3.1 in promoting growth of prostate cancer cells, even while AR is inhibited. We see additive effects of AR inhibition via enzalutamide, and a partial rescue of the growth inhibition seen with AR antagonism and androgen depletion when NKX3.1 is overexpressed. This suggests a differential role for NKX3.1, perhaps during tumor initiation or early stages of disease versus the dependency on expression when AR activity is high in late stages and in castration-resistant disease.

In the literature, there is disagreement concerning the role of NKX3.1 during PCa, as there is evidence for both tumor-suppressive and oncogenic functions. NKX3.1 has a long-established role as being tumor suppressive in mouse models and in early stages of disease. In early-stage disease, NKX3.1 has been shown to be protective by promoting DNA damage responses [34,35,36,37,38], and outside of its role as a transcription factor, NKX3.1 has been shown to play a role in mitochondria in controlling oxidative damage and reducing oxidative stress [39,40]. However, regarding PCa progression, there appears to be a pro-survival benefit attributed to maintained NKX3.1 expression or overexpression, especially in late-stage disease. Its contribution to PCa initiation and progression appears modular, and there is growing evidence that traditional tumor suppressors (such as PTEN and p27^KIP1^) can assume oncogenic roles in cancer [41,42]. And while it widely known that NKX3.1 undergoes loss of heterozygosity because of deletions to the short arm of chromosome 8 [14,21], this event is not a focal deletion as is seen with traditional PCa tumor suppressors such as *PTEN* and *TP53* [21].

AR itself shows a similar pattern of stage- and context-dependent tumor suppression in prostate cancer. AR switches from tumor suppressive to tumor promoting during prostate carcinogenesis [4,43,44], and AR is still the driving oncogene in the majority of late-stage PCa [2,6,21,45]. Similarly, NKX3.1 expression is still maintained throughout the course of the disease and metastatic spread [23], and in the AR-positive and AR-driven [6] prostate cancer cell line models commonly employed in PCa research (Figure 1). The relationship between NKX3.1 expression and advanced stage disease is mediated directly by AR and its activity, and the cooperativity between NKX3.1 and AR was previously found to be highly androgen-dependent: both forming a complex on chromatin and activating transcription of target genes including NKX3.1 and AR themselves [9]. Both here and previously [33], we have illustrated that NKX3.1 may be regulating only a subset of sARE-regulated AR-target genes. sAREs seem to drive differentiation and are potentially tumor suppressive in early-stage disease [33], as inability to activate an sARE leads to incomplete virilization in male mice [46] and produces a more oncogenic AR [47]. sAREs are also strongly regulated by co-factors, such as NKX3.1 [6,33], as these sites allow greater specificity and response, in part due to cooperative interactions with AR itself [48] or with other transcription factors [49]. However, these genes are also enriched for binding of oncogenes like FOXA1 [50], and recent work has suggested that in late-stage disease, these sites may be more tumor-promoting, in that they can be occupied by an AR monomer during androgen deprivation [51], and co-factors can drive oncogenic activity off of the sAREs [52], with cAREs actually being more tumor suppressive. All these data are in line with the work presented here illustrating an oncogenic role of NKX3.1 through an interaction with AR. Additionally, differential isoform expression, as Figure 1B suggests, may play a role in different stages of disease, throughout initiation, castration resistance, and, finally, AR antagonist resistance. Future work will be dedicated to dissecting and examining how AR and NKX3.1 are functioning in late-stage and therapy-resistant disease as compared to early in carcinogenesis and how we can selectively target their interaction and activity therapeutically.

Lending further credence to its oncogenic activity, NKX3.1 expression has been correlated with stem-like features that may function to promote aggressive forms of PCa [53]. Furthermore, NKX3.1 can substitute for the Yamanaka factor OCT4, a transcription factor belonging to POU class of homeobox genes, and it is sufficient to facilitate cellular reprogramming of differentiated cells back to pluripotent stem cells [54]. We and others have identified other members of the pluripotent stem cell transcriptional circuitry [55] as being important AR-regulated oncogenes in prostate cancer, including SOX2 [25,56,57,58] and NANOG [59,60]; however, we rarely see these co-expressed in cancer, or in particular, expressed with OCT4 [25]. These findings may suggest that NKX3.1 can play a role in mediating the stem-like oncogenic features of prostate cancer cells by interacting with these aberrantly expressed stem-cell transcription factors in late-stage disease. This may provide an explanation to the discrepancies seen with NKX3.1’s role in early-stages of prostate cancer, given that factors like SOX2 are AR-repressed [25] and upregulated after androgen targeted therapy and promote therapeutic resistance [25,56,57,58]. Further work is necessary to elucidate how NKX3.1 mediates stem cell-like phenotypes and interacts with other transcription factors outside of AR and FOXA1.

## 5. Conclusions

The canonical role of NKX3.1 as a tumor suppressor is examined in late-stage prostate cancer models. Here, we illustrate that NKX3.1 has an oncogenic role in promoting the survival of prostate cancer. We show that NKX3.1 is co-expressed and interacts with AR and FOXA1 in AR-positive prostate cancer cells, both sensitive and resistant to the AR antagonist enzalutamide. Knockdown of NKX3.1 protein and mRNA, with two different siRNAs, leads to a decrease in survival in every NKX3.1-positive cell line examined, no matter what hormonal context, and decreases canonical AR target gene activity. Finally, overexpression experiments revealed that NKX3.1 is sufficient to promote therapy-resistant growth by decreasing reliance on androgens as well as reducing the anti-proliferative effects of AR antagonism with enzalutamide. These effects coincide with an increase in selective androgen-regulated genes and a decrease in expression of canonical AR targets such as PSA. Taken together, these data illustrate that NKX3.1 is necessary and sufficient for the survival of prostate cancer cells, thereby solidifying its oncogenic role in these late-stage and AR-targeted therapy-resistant models of disease.

## Figures and Tables

**Table 1:** Primer Sequences Used for RT-PCR Analysis of Genes.

Gene	Forward Primer (5′ to 3′)	Reverse Primer (5′ to 3′)
AR Exon 1-2 (Total AR)	ATCCCAGTCCCACTTGTGTC	GGTCTTCTGGGTGGAAAGT
AR Exon 4 (Full Length, FL)	CGGAAGCTGAAGAAACTTGG	ATGGCTTCCAGGACATTCAG
AR Variant 7 (AR-V7)	CCATCTTGTCGTCTTCGGAAATGTTATGA	TTTGAATGAGGCAAGTCAGCCTTTCT
β-actin (*ACTB*)	CACCATTGGCAATGAGCGGTTC	AGGTCTTTGCGGATGTCCACGT
*ERG*	CGCAGAGTTATCGTGCCAGCAGAT	CCATATTCTTTCACCGCCCACTCC
*FKBP5*	TCTCATGTCTCCCCAGTTCC	TTCTGGCTTTCACGTCTGTG
*GAPDH*	GTCTCCTCTGACTTCAACAGCG	ACCACCCTGTTGCTGTAGCCAA
GR (*NR3C1*)	TCTGAACTTCCCTGGTCGAA	GTGGTCCTGTTGTTGCTGTT
*IGFBP3*	AAAAGCAGTGTCGCCCTTC	TAGCAGTGCACGTCCTCCTT
*NKX3-1*	CAGTCCCTACTGAGTACTCTTTCTCTC	CACAGTGAAATGTGTAATCCTTGC
PSA (*KLK3*)	TCATCCTGTCTCGGATTGTG	ATATCGTAGAGCGGGTGTGG
SARG (*C1orf116*)	AGTCTGAGCCAGCCACAACT	TGTGGATATTCCTAGGGAGG
*TMPRSS2*	TGTGGTCCCTTCCAATGCTGTG	TGCTCATGGTTATGGCACTTGGC

## Data Availability

All raw data from plots and Western Blots are available upon request.

## Supplementary Materials

The following supporting information can be downloaded at: https://www.mdpi.com/article/10.3390/cancers17020306/s1, Figure S1: NKX3.1 siRNA annealing sites and complementarity; Figure S2: mRNA fold change by RT-qPCR; Figure S3: Cell viability time course of PCa cell lines post siRNA transfection; Figure S4: Protein levels 3 days post-transfection with siRNA; Figure S5: mRNA levels of AR Exon 4 (full-length) and AR V7 in enzalutamide-sensitive and enzalutamide-resistant PCa cell lines; Figure S6: Cell viability time course and protein levels of endogenous and overexpressed NKX3.1 protein in LNCaP cells.
