# Supplementary material for: The Homeobox Transcription Factor NKX3.1 Displays an Oncogenic Role in Castration-Resistant Prostate Cancer Cells
Source: Cancers (Basel). 2025 Jan 18;17(2):306. doi: 10.3390/cancers17020306 (PMC11763476; doi:10.3390/cancers17020306)
Supplement: Supplementary file 1 [file cancers-17-00306-s001.zip › cancers-3390649-supplementary.pdf]

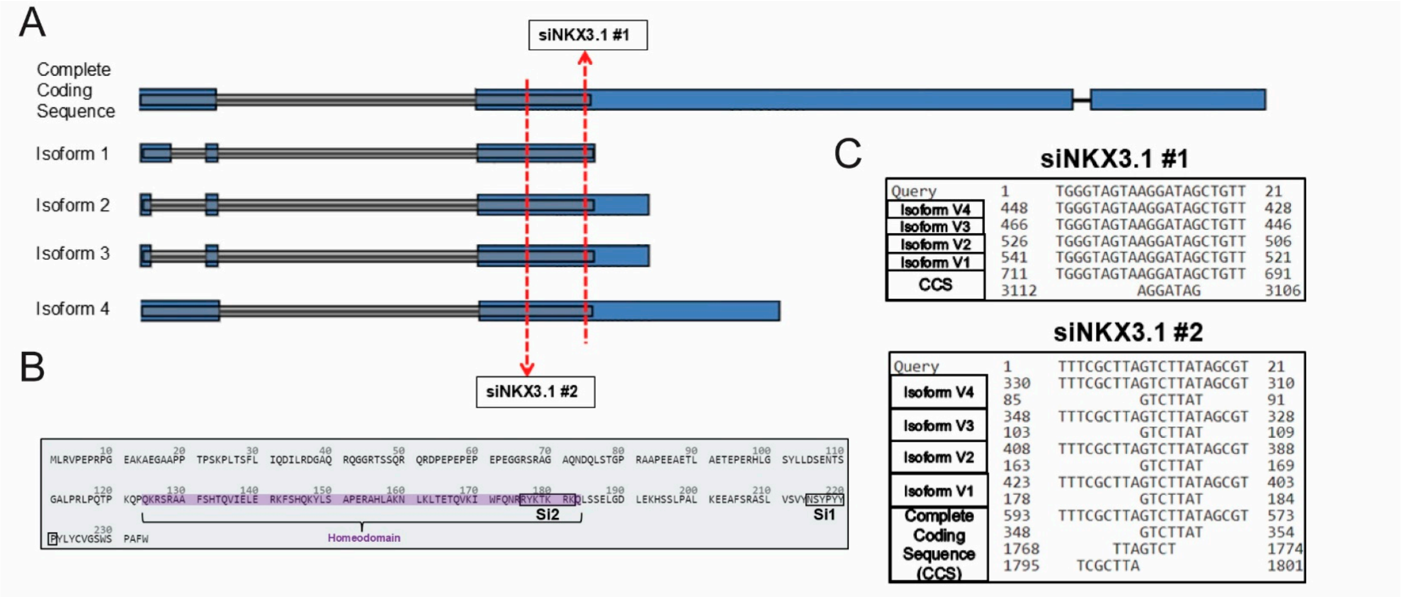

Supplementary Figure S1. NKX3.1 siRNA annealing sites and complementarity. A) Schematic of the canonical complete coding sequence of NKX3.1 and four variant isoforms. Blue bars denote exons, grey boxes denote introns, and the homeodomain is boxed within blue exon bar. B) The 234 amino acid (AA) length of canonical NKX3.1 with the homeodomain highlighted in purple. The seven AA target of siNKX3.1 #1 and #2 are shown. C) The siRNA sequence of siNKX3.1 #1 and #2 labeled as "query'" and all complementary sequences (7 base pairs and up) of NKX3.1 and its four isoforms.

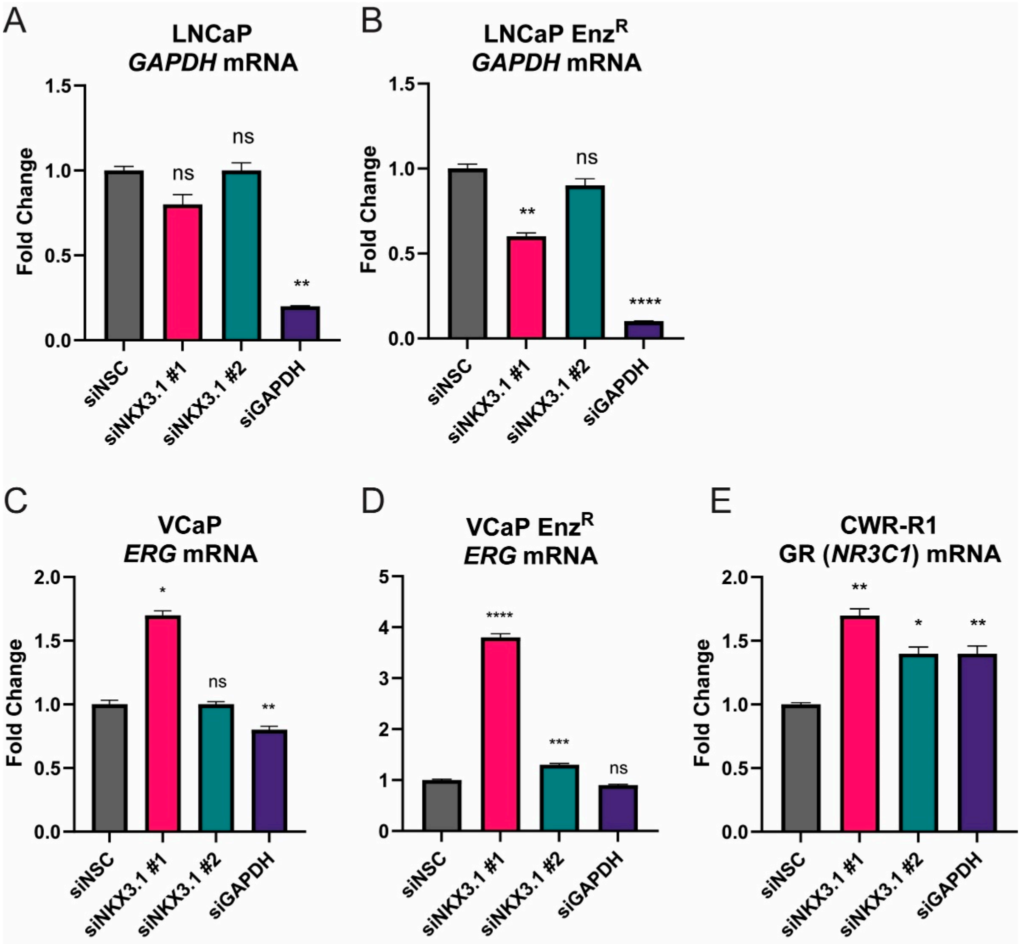

Supplementary Figure S2. mRNA fold change by RT-qPCR. A) *GAPDH* mRNA levels surveyed by RT-qPCR in LNCaP cells. B) *GAPDH* mRNA levels surveyed by RT-qPCR in LNCaP Enz^{R} cells. C) ERG mRNA levels surveyed by RT-qPCR in VCaP cells. D) *ERG* mRNA levels surveyed by RT-qPCR in VCaP Enz^{R} cells. E) GR (*NR3C1*) mRNA levels surveyed by RT-qPCR in CWR-R1 cells. Statistical significance was determined by students t-test relative to siNSC control. The threshold for statistical significance set as follows: *ns p*>0.05, * *p* < 0.05, ** *p* < 0.01, *** *p*<0.001, **** *p*<0.0001.

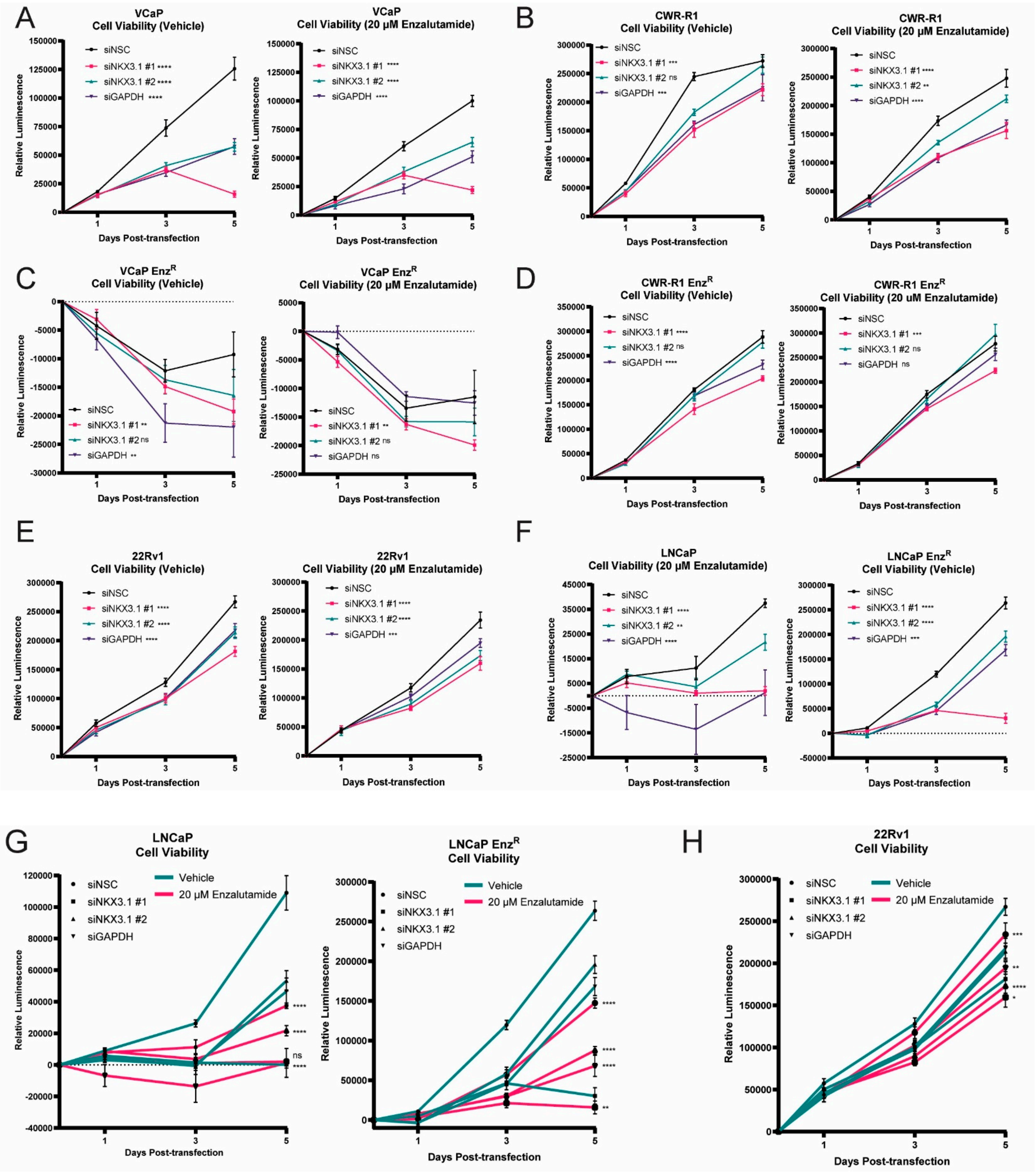

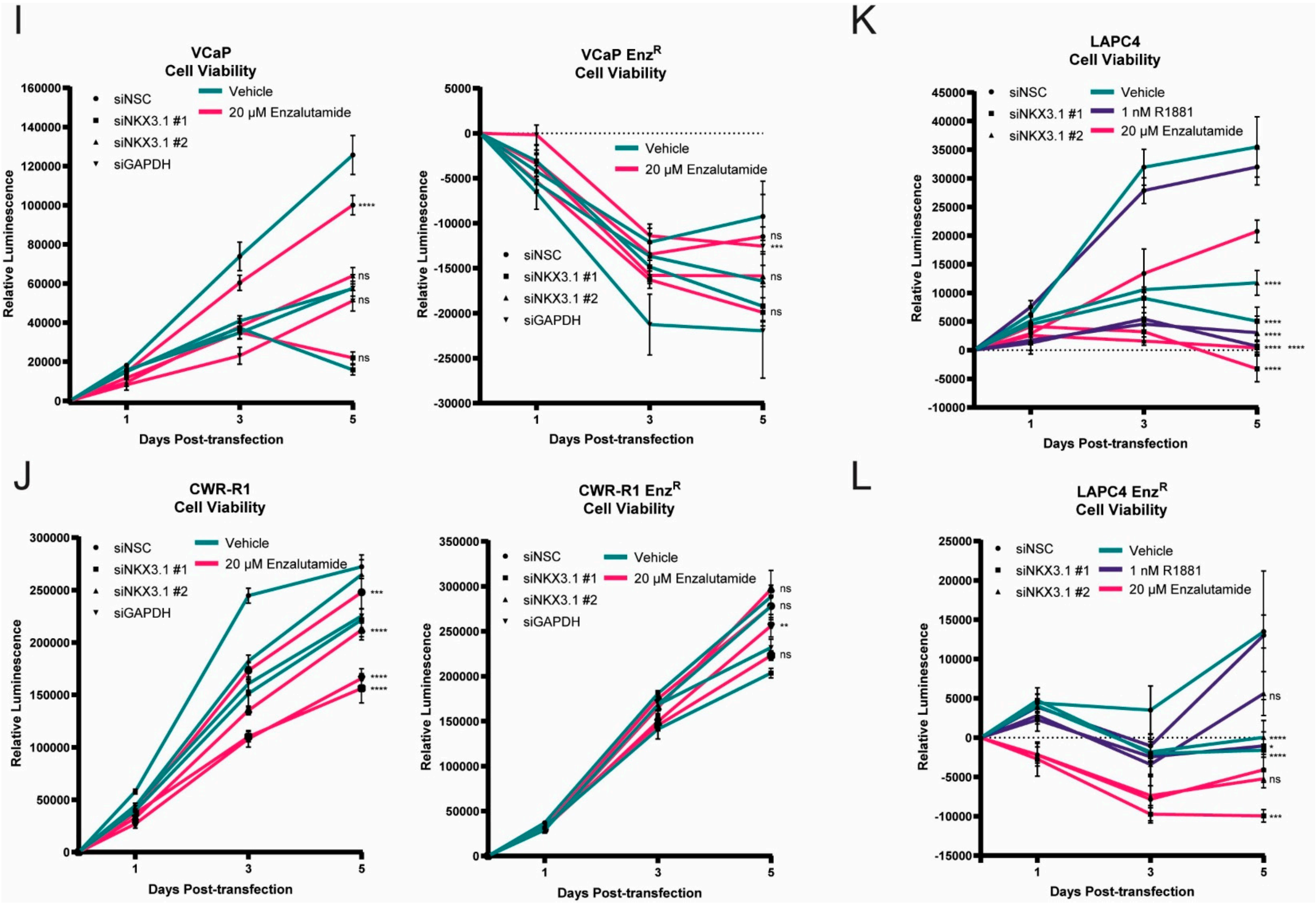

Supplementary Figure S3. Cell viability time course of PCa cell lines post siRNA transfection. A) Cell viability of VCaP cells transfected with siRNA and treated with vehicle or 20 µM enzalutamide over 5 days. B) Cell viability of CWR-R1 cells transfected with siRNA and treated with vehicle or 20 µM enzalutamide over 5 days. C) Cell viability of VCaP Enz^R cells transfected with siRNA and treated with vehicle or 20 µM enzalutamide over 5 days. D) Cell viability of CWR-R1 Enz^R cells transfected with siRNA and treated with vehicle or 20 µM enzalutamide over 5 days. E) Cell viability of 22Rv1 cells transfected with siRNA and treated with vehicle or 20 µM enzalutamide over 5 days. F) Cell viability of LNCaP and LNCaP Enz^R cells transfected with siRNA and treated with vehicle and 20 µM enzalutamide (respectively). G) Effect of enzalutamide on cell viability of LNCaP and LNCaP Enz^R cells. H) Effect of enzalutamide on cell viability of 22Rv1 cells. I) Effect of enzalutamide on cell viability of VCaP and VCaP Enz^R cells. J) Effect of enzalutamide on cell viability of CWR-R1 and CWR-R1 Enz^R cells. K) Cell viability LAPC4 cells transfected with siRNA and treated with vehicle, 1 nM R1881, or 20 µM enzalutamide. L) Cell viability LAPC4 Enz^R cells transfected with siRNA and treated with vehicle, 1 nM R1881, or 20 µM enzalutamide. Statistical significance was determined by one-way *ANOVA* and relative to siNSC control (**Figures A-F, K, L**). Statistical significance of each siRNA was determined by two-way *ANOVA* relative to vehicle (**Figures G-J)**. The threshold for statistical significance set as follows: *ns* $p>0.05$, * $p < 0.05$, ** $p < 0.01$, *** $p<0.001$, **** $p<0.0001$.

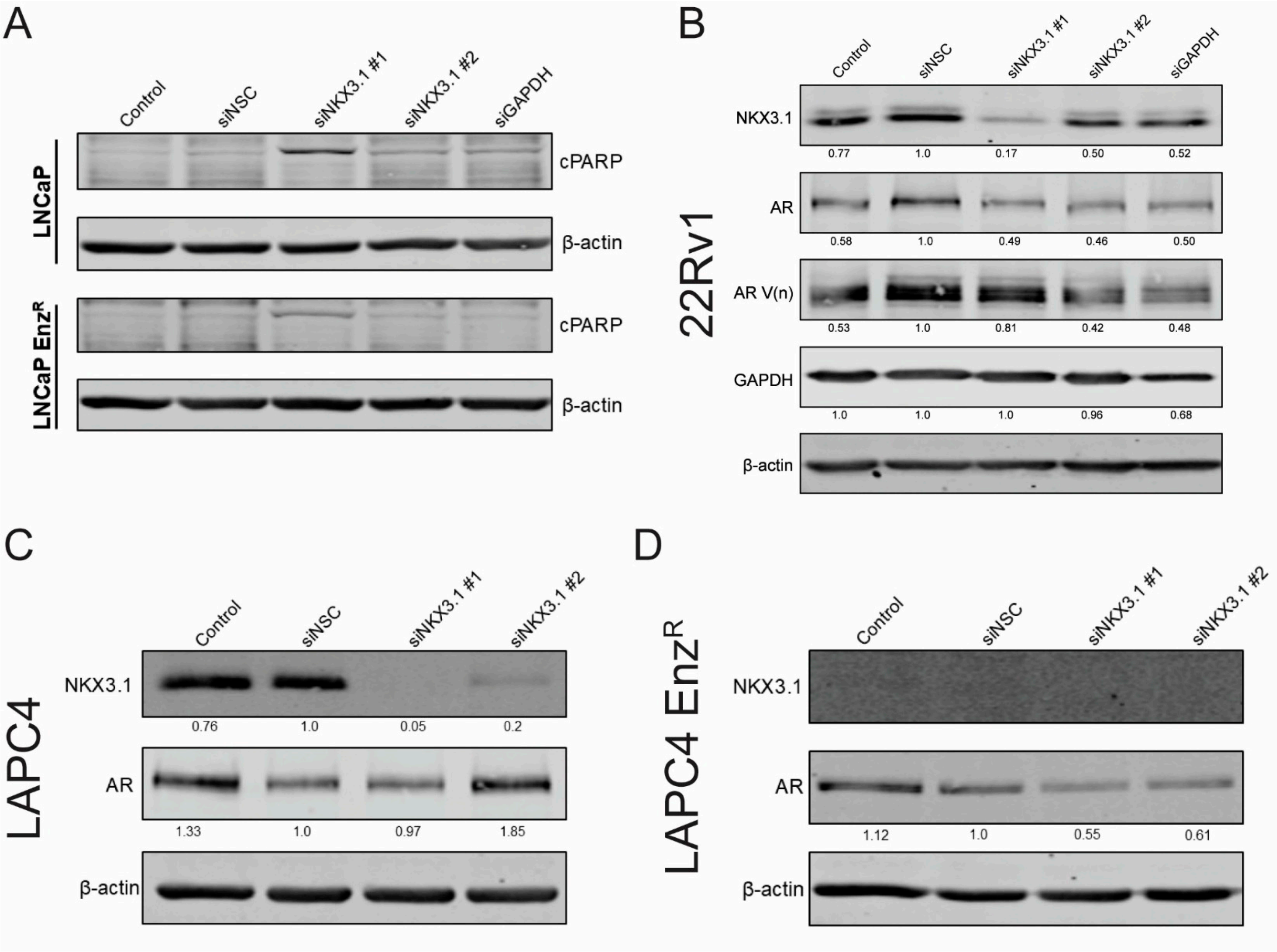

Supplementary Figure S4. Protein levels 3-days post-transfection with siRNA. A) Western blot depicting cPARP protein levels in LNCaP and LNCaP Enz^R cells. β-actin shown as a loading control. B) Western blot showing protein levels in 22Rv1 cells 3 days after transfection. Protein bands quantified relative to β-actin. C) Western blot showing protein levels in LAPC4 cells 3 days post transfection. Protein bands quantified relative to β-actin. D) Western blot showing protein levels in LAPC4 Enz^R cells 3 days post transfection. Protein bands quantified relative to β-actin.

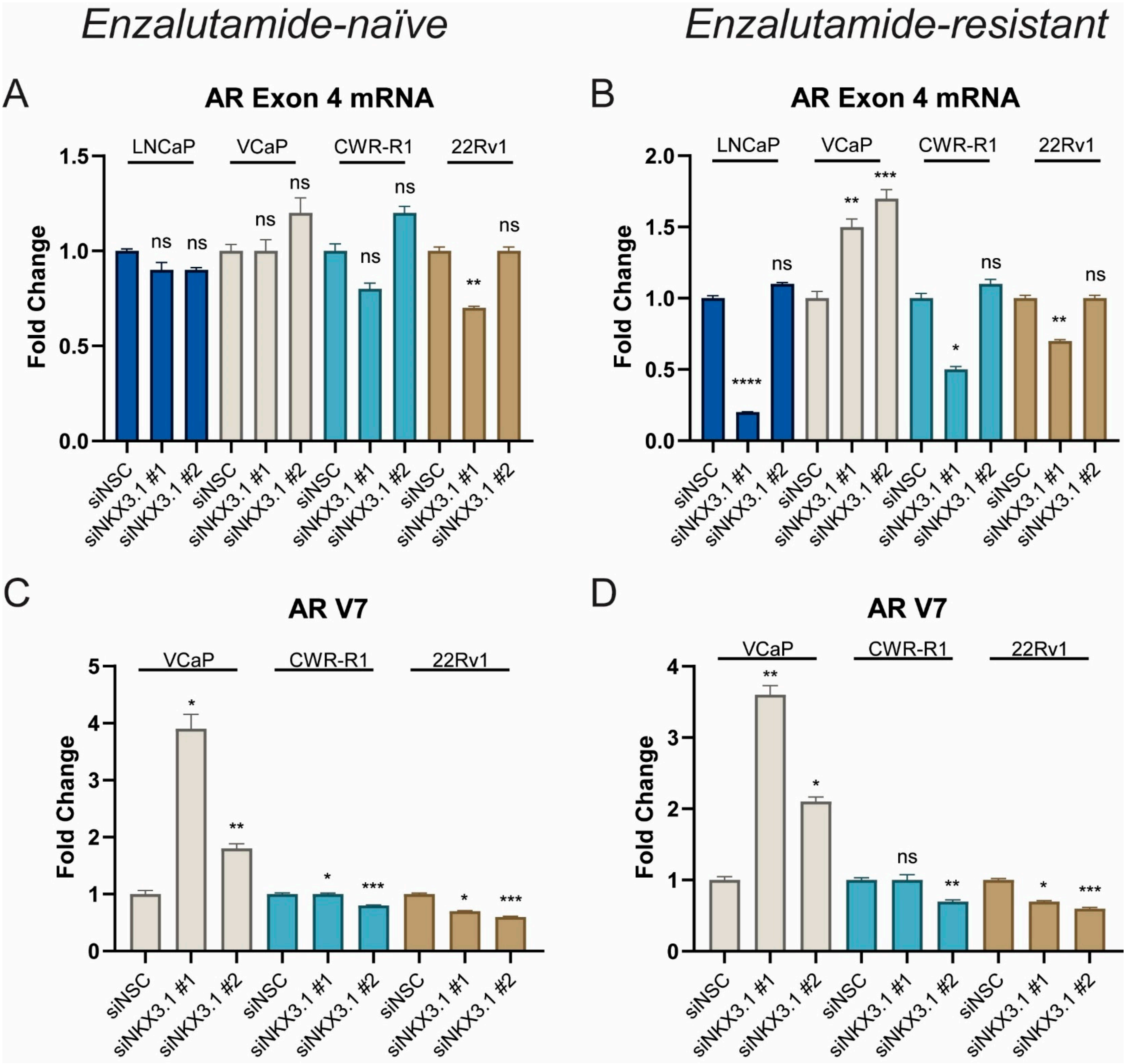

Supplementary Figure S5. mRNA levels of AR Exon 4 (full-length) and AR V7 in enzalutamide-sensitive and enzalutamide-resistant PCa cell lines. A) AR Exon 4 (full-length) mRNA levels in enzalutamide-naïve cell lines. Statistical significance determined by student's t-test and relative to siNSC control for each individual cell lines. B)) AR Exon 4 (full-length) mRNA levels in enzalutamide-resistant cell lines. Statistical significance determined by student's t-test and relative to siNSC control for each individual cell line. C) AR V7 mRNA levels in enzalutamide-naïve cell lines. Statistical significance determined by student's t-test and relative to siNSC control for each individual cell line. D) AR V7 mRNA levels in enzalutamide-resistant cell lines. Statistical significance determined by student's t-test and relative to siNSC control for each individual cell line. The threshold for statistical significance is set as follows: *ns* $p>0.05$, * $p < 0.05$, ** $p < 0.01$, *** $p<0.001$, **** $p<0.0001$.

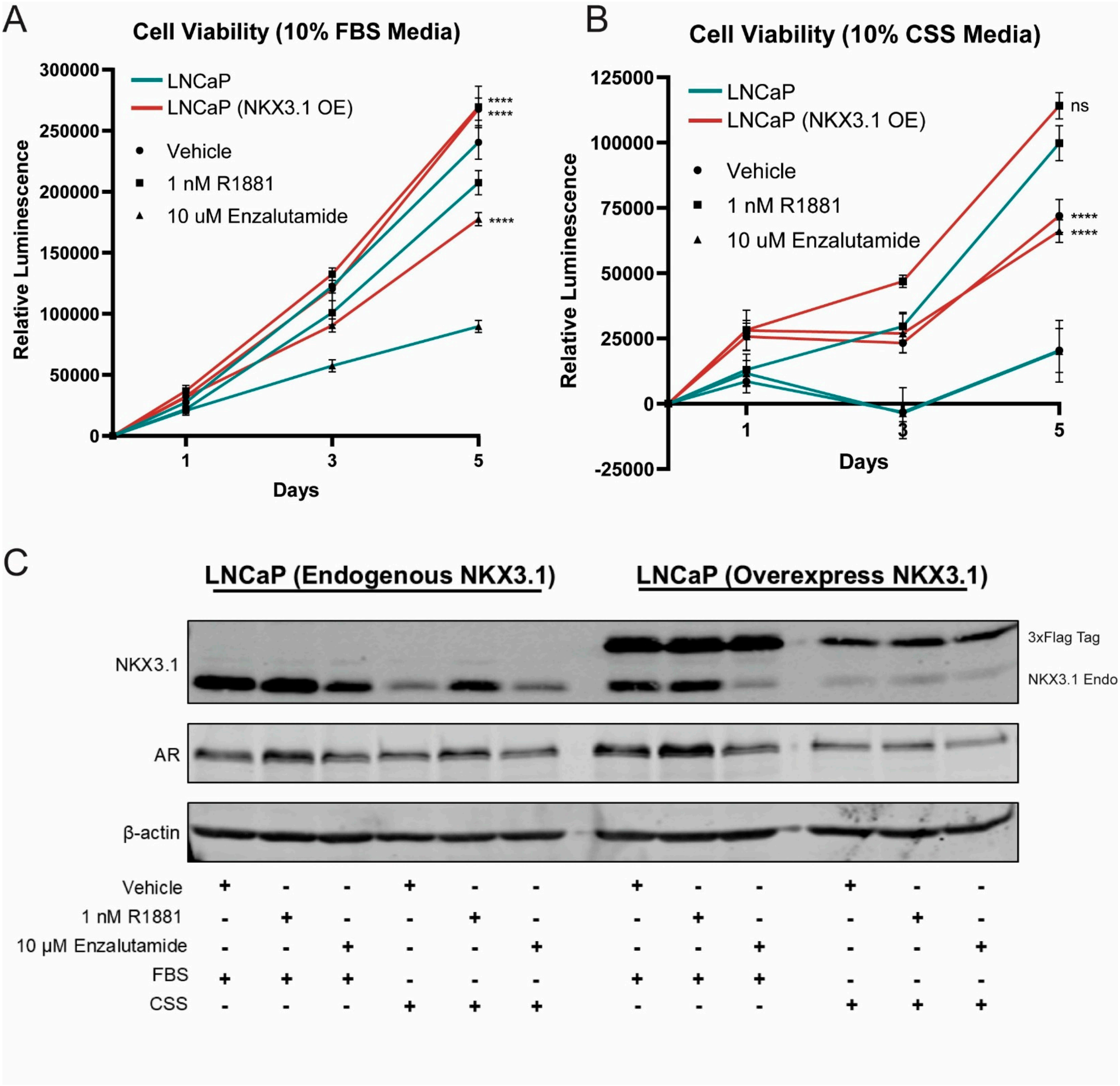

Supplementary Figure S6. Cell viability time course and protein levels of endogenous and overexpressed NKX3.1 protein in LNCaP cells. A) Cell viability time course of parental LNCaP cells and 3x-Flag tagged NKX3.1-expressing LNCaP cells grown in 10% FBS media and treated with vehicle, 1 nM R1881, or 10 μM enzalutamide. B) Cell viability time course of parental LNCaP cells and 3x-Flag-tagged-NKX3.1-expressing LNCaP cells (NKX3.1 OE) grown in 10% CSS media and treated with vehicle, 1 nM R1881, or 10 μM enzalutamide. C) Protein levels in LNCaP parental cells and 3x-Flag-tagged-NKX3.1-expressing LNCaP cells grown in 10% FBS media or 10% CSS media and treated with vehicle, 1 nM R1881, or 10 μM enzalutamide. The threshold for statistical significance is set as follows: *ns* $p>0.05$, * $p < 0.05$, ** $p < 0.01$, *** $p<0.001$, **** $p<0.0001$.
